# Analgesic Efficacy of Celecoxib Versus Ibuprofen After Third Molar Surgery: A Systematic Review and Meta-Analysis of Randomized Controlled Trials

**DOI:** 10.7759/cureus.116000

**Published:** 2026-09-09

**Authors:** Dana Saleh Tumaiheer, Rawaa M Buloushi, Farah Ahmad Alenezi, Zahraa Bader Khasroh, Maryam S J AlNeamah, Dhari Mohammed Alhajri, Maryam Bader Alzamanan, Ahmed Abdelaziz

**Affiliations:** 1 Department of Dentistry, Sabahiya West Healthcare Centre, Al-Ahmadi, KWT; 2 Department of Dentistry, AlQairawan Health Center, Al-Jahra, KWT; 3 Department of Dentistry, Kuwait Ministry of Health, Kuwait City, KWT; 4 Department of Biostatistics, Faculty of Medicine Al-Azhar University, Cairo, EGY

**Keywords:** celecoxib, meta-analysis, nonsteroidal anti-inflammatory medications (nsaids), pain severity, third-molar surgery

## Abstract

Third-molar surgery is commonly associated with acute postoperative pain, for which nonsteroidal anti-inflammatory drugs (NSAIDs) are widely used. Celecoxib, a selective cyclooxygenase-2 inhibitor, may provide effective analgesia while potentially offering improved tolerability compared with conventional nonselective NSAIDs. We performed a systematic review and meta-analysis of randomized trials that compared celecoxib with ibuprofen following surgical extraction of the impacted mandibular third molars. Scopus, PubMed, Web of Science (WoS), and the Cochrane Central Register of Controlled Trials were searched from inception to August 2026. Ten RCTs involving 1,090 patients were included, with 671 patients receiving celecoxib and 419 receiving ibuprofen. Postoperative pain was evaluated at 4-8, 12-16, and 24 hours using the visual analog scale (VAS), total pain relief (TOPAR/TOTPAR), and sum of pain relief and pain intensity difference (SPRID/SPID). No significant differences were observed between celecoxib and ibuprofen at 4-8 or 12-16 hours across any of the pain assessment scales. At 24 hours, celecoxib was associated with significantly lower VAS pain scores (standardized mean difference [SMD], −0.35; 95% CI, −0.61 to −0.08; p = 0.01), whereas no significant difference was observed using TOPAR/TOTPAR (SMD, 0.36; 95% CI, −0.00 to 0.73; p = 0.05). Rescue analgesic use did not differ significantly between groups (RR, 1.00; 95% CI, 0.80-1.25; p = 1.00) and neither did adverse events (RR, 0.93; 95% CI, 0.65-1.32; p = 0.67). Trial sequential analysis did not provide conclusive evidence of a beneficial effect. Overall, celecoxib and ibuprofen demonstrated broadly comparable analgesic efficacy following third-molar surgery. Although celecoxib may provide a modest reduction in pain intensity at 24 hours, current evidence does not establish consistent overall superiority over ibuprofen.

## Introduction and background

Third-molar extraction is considered one of the most common oral surgical procedures and is commonly followed by acute postoperative pain, swelling, and limited oral function [1]. The inflammatory response associated with surgical tissue injury is a major contributor to postoperative pain, particularly during the first several hours after extraction [2]. Effective analgesia is therefore an important component of postoperative care, allowing patients to resume normal activities and oral intake while minimizing the need for rescue medication [3].

Nonsteroidal anti-inflammatory drugs (NSAIDs) are widely used as first-line analgesics following third-molar surgery because of their ability to inhibit prostaglandin synthesis and reduce inflammation [4]. Ibuprofen is among the most commonly prescribed NSAIDs in this setting and has demonstrated reliable analgesic efficacy [5]. However, its nonselective inhibition of cyclooxygenase (COX)-1 and COX-2 may be associated with gastrointestinal adverse effects and other NSAID-related complications, particularly with repeated dosing [5]. Celecoxib, a selective COX-2 inhibitor, has emerged as a potential alternative because it provides anti-inflammatory and analgesic effects while producing less COX-1 inhibition [6]. This pharmacological distinction may offer advantages in terms of tolerability while maintaining adequate postoperative pain control [6].

Previous systematic reviews have established the role of NSAIDs in the management of acute postoperative dental pain, with evidence supporting their overall analgesic efficacy following dental surgery [5,7,8]. However, the available literature has largely evaluated NSAIDs as a broader therapeutic class or compared individual agents with placebo or other analgesics, providing less clarity regarding the relative efficacy of selective COX-2 inhibition versus conventional nonselective NSAID therapy after third-molar surgery [8].

Thus, this paper aimed to synthesize the available randomized controlled evidence comparing celecoxib with ibuprofen for postoperative pain management following third-molar surgery.

## Review

Methods

We conducted this systematic review and meta-analysis according to the Preferred Reporting Items for Systematic Reviews and Meta-Analyses (PRISMA) guidelines [9] and the guidelines of the Cochrane Handbook for Systematic Reviews of Interventions [10].

Literature Search and Screening

We conducted an electronic search of PubMed, Scopus, Web of Science (WoS), and the Cochrane Central Register of Controlled Trials (CENTRAL) from inception to August 2026 to identify randomized controlled trials (RCTs) comparing celecoxib with ibuprofen for postoperative pain following third-molar surgery. The search strategy included terms related to celecoxib, ibuprofen, NSAIDs, COX-2 inhibitors, third-molar surgery or extraction, and postoperative pain or analgesia. The detailed strategy for each database is shown in the Appendix.

We also performed backward and forward citation searching of the references of relevant articles and retrieved studies to identify additional eligible trials. Titles and abstracts were screened initially, followed by full-text assessment of potentially eligible studies according to the prespecified eligibility criteria.

Eligibility Criteria

Studies were considered for inclusion according to the following PICO criteria:

Population (P): adults undergoing surgical extraction of the impacted mandibular third molars.

Intervention (I): celecoxib administered for postoperative pain management at any dose or dosing regimen.

Comparator (C): ibuprofen administered for postoperative pain management at any dose or dosing regimen.

Outcomes (O): postoperative pain, analgesic efficacy, rescue analgesic use, and adverse events.

Only RCTs that directly compared celecoxib with ibuprofen and reported at least one prespecified outcome were considered eligible. Studies were excluded if they were not RCTs, did not involve surgical extraction of impacted third molars, did not directly compare celecoxib with ibuprofen, or did not report any of the prespecified outcomes. Reviews, meta-analyses, observational studies, case reports, conference abstracts without sufficient data, and studies with unavailable full texts were also excluded.

Outcomes

The primary outcomes were postoperative pain scores at 4-8, 12-16, and 24 hours, assessed using the visual analog scale (VAS) [11]. The VAS is a specific measure of pain intensity consisting of a 100-mm line anchored by “no pain” (0 mm) and “worst pain imaginable” (100 mm). Additional measures of analgesic efficacy included total pain relief (TOPAR/TOTPAR), calculated as the time-weighted sum of pain relief scores [12], and the sum of pain relief and pain intensity difference (SPRID/SPID), calculated as the time-weighted sum of pain relief and pain intensity difference scores [13]. Secondary outcomes included the number of patients requiring rescue analgesic medication and the number of patients experiencing adverse events.

Quality Assessment and Data Extraction

The risk of bias of the included RCTs was assessed using the Cochrane Risk of Bias 2 (RoB 2) tool [14]. The tool evaluates five domains: randomization process, deviations from the intended interventions, missing outcome data, measurement of the outcome, and selection of the reported result. Each domain was classified as some concerns, a low risk of bias, or a high risk of bias. Disagreements between reviewers were resolved through discussion and consensus with the corresponding author.

Data were extracted using a standardized Excel data extraction form (Microsoft Corporation, Redmond, WA, USA). The information included study characteristics and patient demographics, including country, sample size, mean age, sex distribution, celecoxib and ibuprofen dosing regimens, eligibility criteria, surgical characteristics, assessed outcomes, and key findings. Information regarding risk of bias and outcome data was also extracted.

Statistical analysis

Dichotomous outcomes were extracted as events and total patients and were pooled as risk ratios (RRs) with 95% confidence intervals (CIs). Continuous outcomes were extracted as means, standard deviations (SDs), and sample sizes and were pooled as standardized mean differences (SMDs) with 95% CIs. All pooled estimates were calculated using a restricted maximum likelihood (REML) random-effects model. REML was used to estimate the between-study variance (τ²), while the random-effects model accounts for both within-study sampling error and between-study heterogeneity, thereby allowing for variation in the underlying treatment effects across studies. Subgroup analyses were performed according to the pain assessment scale used in the included studies to explore potential differences in treatment effects across measurement scales. Statistical heterogeneity was assessed using the I² statistic and the chi-square test, with an I² value of ≥50% considered indicative of substantial heterogeneity.

Trial sequential analysis (TSA) was performed to assess the conclusiveness and robustness of the cumulative evidence and to determine whether the available information size was sufficient to confirm or refute the observed treatment effect. TSA combines cumulative meta-analysis with sequential monitoring boundaries to control the risk of random errors associated with repeated significance testing as additional studies are added to a meta-analysis. The required information size represents the estimated amount of information needed to draw a sufficiently reliable conclusion, while sequential monitoring boundaries are used to determine whether the cumulative evidence has crossed a threshold for benefit, harm, or futility before reaching the required information size. TSA was therefore used to determine whether the observed effects were sufficiently supported by the accumulated evidence or whether additional trials would be required.

All statistical analyses were performed using Stata/MP version 19 (StataCorp, College Station, TX, USA). A p-value of <0.05 was considered statistically significant.

Results

Literature Search and Study Selection

Our search yielded 1,989 records. After duplicates and title and abstract screening, 29 articles were considered potentially eligible and retrieved for full-text assessment. One article could not be retrieved, leaving 28 articles for full-text screening. Ultimately, 10 RCTs were included in the quantitative analysis [15-24]. The selection process is provided in the PRISMA flow diagram in Figure 1.

**Figure 1:**
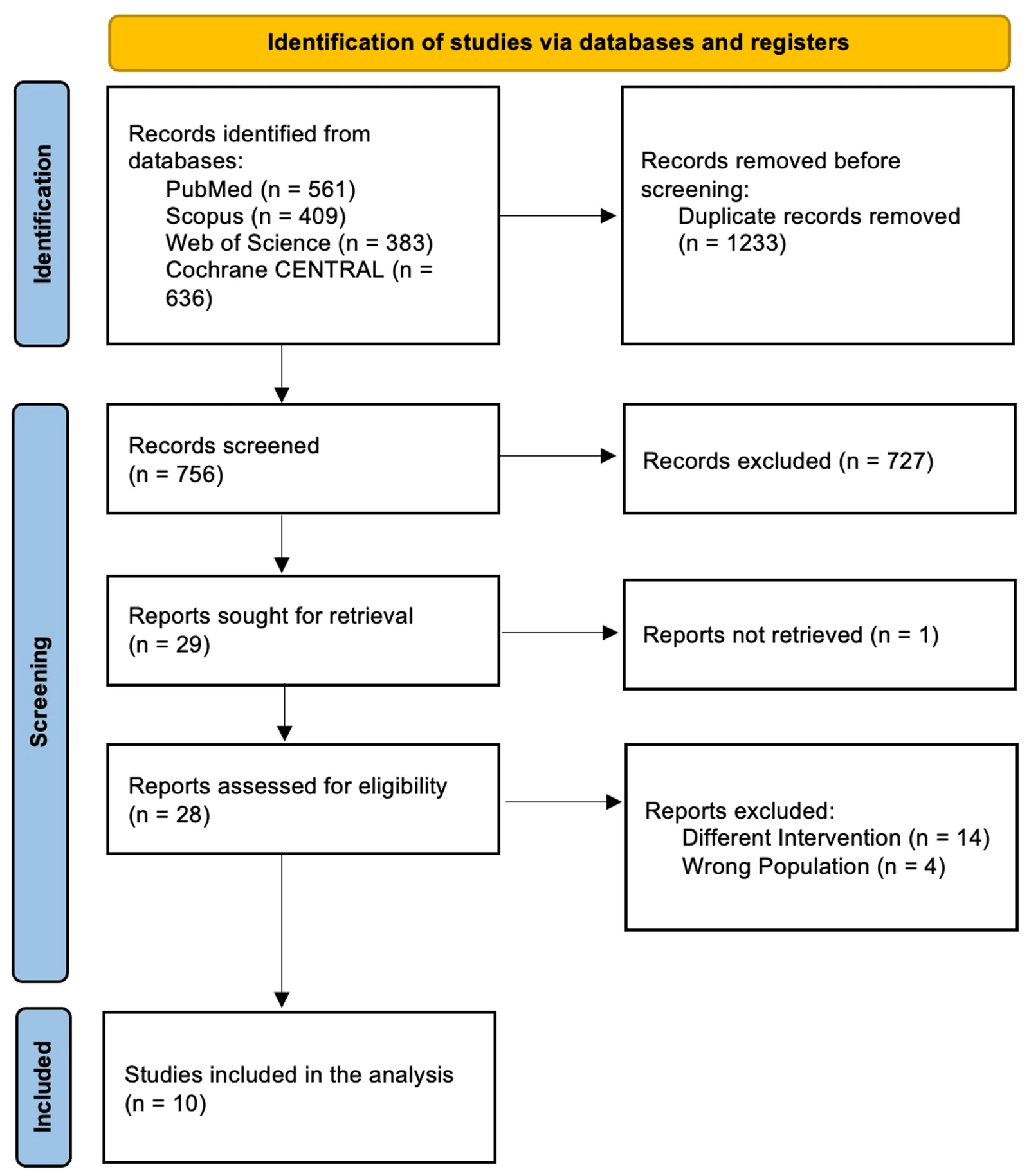
PRISMA flow diagram PRISMA, Preferred Reporting Items for Systematic Reviews and Meta-Analyses

Study Characteristics and Quality Assessment

A total of 10 RCTs were included, conducted in Nigeria, the United States, Saudi Arabia, Italy, Iran, and Finland between 1999 and 2024. The studies included 1,090 patients, of whom 671 (61.5%) were allocated to celecoxib and 419 (38.5%) to ibuprofen. Approximately half of the participants were female, and the mean age across studies ranged from 21.1 ± 4.8 to 30.3 ± 5.5 years. Celecoxib dosing regimens varied across studies and included a 400-mg loading dose followed by 200 mg, a single 400-mg dose, preemptive administration of 200 mg, 200 mg twice daily for five days, and 100 mg twice daily for three days. Ibuprofen was administered at doses of 400 or 600 mg. Detailed characteristics of the included studies and baseline patient data are presented in Tables 1, 2.

**Table 1:** Summary characteristics of the included studies. NR, not reported; POD, postoperative day; PID, pain intensity difference; PGE₂, prostaglandin E₂; PR, pain relief; PRID, pain relief intensity difference; RCT, randomized controlled trials; SD, standard deviation; SPID, sum of pain intensity difference; SPRID, sum of pain relief and pain intensity difference; SPID8, 8-hour sum of pain intensity differences; SPID12, 12-hour sum of pain intensity differences; TOPAR, total pain relief; TOPAR8, 8-hour total pain relief; TOPAR12, 12-hour total pain relief; TXB₂, thromboxane B₂; VAS, visual analog scale

Study	Country	Study design	Patients (celecoxib/ibuprofen)	Celecoxib dose and regimen	Ibuprofen dose and regimen	Rescue medication	Surgical procedure	Outcomes
Akinbade et al., 2018 [15]	Nigeria	RCT	90 (45/45)	400 mg loading, then 200 mg 12 hourly	400 mg 8‑hourly	NR	Extraction under 2% lignocaine ﻿hydrochloride with ﻿1:100,000 adrenaline; three‑sided flap, bone removal, tooth sectioning if needed; same operator	Postoperative pain (VAS at 4, 8, 16, 24, 48 h); adverse effects; discontinuation rates
Al-Sukhun et al., 2012 [16]	Finland	RCT	93 (48/45)	200 mg pre‑emptively 1 h before surgery	400 mg pre‑emptively 1 h before surgery	Paracetamol 1000 mg	Surgical removal under local anesthesia (2% lignocaine with 1:80,000 epinephrine) by the same surgeon; surgery time and difficulty recorded	TOPAR8 (primary), TOPAR12, time to onset, peak pain relief, duration (time to rescue), global evaluation at 8 and 24 h
Bassyoni, 2024 [17]	Saudi Arabia	RCT	60 (30/30)	100 mg 12‑hourly for 3 days	400 mg 6‑hourly for 3 days	Paracetamol 1 g	Standardized surgical extraction under 2% mepivacaine with epinephrine; incision, flap, bone removal, tooth sectioning, irrigation, suturing; performed by calibrated residents under supervision	Pain (patient‑reported VAS at 1, 6, 12, 24, 48, 72 h; examiner‑reported at POD 2, 4, 7); swelling (Gabka‑Matsumura); trismus (interincisal opening); rescue medication use
Cheung et al., 2007 [18]	USA	RCT	114 (57/57)	400 mg single dose	400 mg single dose	NR	Surgical extraction with bone removal; surgery time and trauma rated moderate/severe	Primary: time to onset (median), PID, PR, PRID, time to rescue; secondary: time to perceptible/meaningful relief, PPR, TOTPAR, adverse events
Doyle et al., 2002 [19]	USA	RCT	148 (74/74)	200 mg single dose (maximum daily dose at that time)	400 mg three times daily (q4h) for 3 doses	NR	Surgical extraction of impacted third molars; same surgical technique	SPRID over 2, 4, 8, 12 h; time to meaningful relief; time to rescue; global evaluation; adverse events
Isola et al., 2019 [20]	Italy	RCT	66 (33/33)	200 mg twice daily for 5 days	400 mg twice daily for 5 days	NR	Surgical extraction under local anesthesia (mepivacaine 2% with epinephrine); same surgeon; mucoperiosteal flap, osteotomy, tooth sectioning, closure	Pain (VAS at 30 min; 2, 6, 12, 24, 48 h; 7, 10 d); swelling (5 facial distances); trismus (maximum mouth opening); adverse events
Khan et al., 2002 [21]	USA	RCT	67 (37/30)	200 mg given 8 h and 1 h preoperatively	600 mg given 8 h and 1 h preoperatively	Ketorolac IV (for placebo) or acetaminophen/codeine (for actives)	Extraction under local anesthesia and sedation; microdialysis probes placed in surgical sites after surgery; samples collected for 4 h	Pain intensity (VAS, categorical); PGE2 and TXB2 levels in inflammatory transudate; time to rescue
Malmstrom et al., 1999 [22]	USA	RCT	137 (91/46)	200-mg single dose	400-mg single dose	Acetaminophen or acetaminophen plus hydrocodone	Surgical extraction with local/systemic anesthesia; surgery time and number of teeth recorded	TOPAR8 (primary), TOPAR12, time to onset (stopwatch), peak pain relief, duration (time to rescue), global evaluation, adverse events
Malmstrom et al., 2002 [23]	USA	RCT	286 (241/45)	400-mg/200-mg single dose	400-mg single dose	Acetaminophen plus hydrocodone	Surgical extraction with local and/or systemic anesthesia; same surgical procedure	TOPAR8 (primary), TOPAR12, SPID8, SPID12, patient's global assessment, time to onset, peak PID/PR, time to rescue, percentage rescued, adverse events
Zamiri et al., 2009 [24]	Iran	RCT	29 (15/14)	200 mg given 8 h and 1 h preoperatively	600 mg given 8 h and 1 h preoperatively	NR	Extraction under local anesthesia (2% lidocaine with epinephrine); same surgeon; straight elevator and forceps; flap and bone removal if needed	Pain severity (VAS at 4 and 8 h); side effects; patient satisfaction

**Table 2:** Baseline characteristics of the included patients. M/F, male/female; NR, not reported; SD, standard deviation; VAS, visual analog scale

Study	Sample size, n		Age, mean (SD)		Sex (M/F)		Baseline pain score, mean (SD)		Pain intensity (moderate/severe)		Surgery duration, min, mean (SD)	
	﻿Celecoxib	﻿Ibuprofen	﻿Celecoxib	﻿Ibuprofen	﻿Celecoxib	﻿Ibuprofen	﻿Celecoxib	﻿Ibuprofen	﻿Celecoxib	﻿Ibuprofen	﻿Celecoxib	﻿Ibuprofen
Akinbade et al., 2018 [15]	45	45	26.56 (6.29)	27.22 (7.13)	16/29	17/28	Pre‑op VAS: 20.07 (NR)	Pre‑op VAS: 17.45 (NR)	NR	NR	22.53 (NR)	27.53 (NR)
Al-Sukhun et al., 2012 [16]	48	45	30.3 (5.5)	29.1 (7.9)	25/23	22/23	NR	NR	NR	NR	NR	NR
Bassyoni, 2024 [17]	30	30	25.87 (5.64)	23.27 (3.89)	NR	NR	NR	NR	NR	NR	25.97 (7.24)	27.17 (12.31)
Cheung et al., 2007 [18]	57	57	21.4 (4.2)	22 (4.7)	27/30	27/30	Baseline VAS: 74.3 (12.5) mm	Baseline VAS: 74.8 (13.2) mm	30/27	30/27	30/27	NR
Doyle et al., 2002 [19]	74	74	21.1 (4.8)	21.8 (6)	30/44	33/41	Baseline VAS: 71.1 (11.9) mm	Baseline VAS: 71 (12.5) mm	47/27	49/25	15.6 (9.2)	13.7 (7.1)
Isola et al. 2019 [20]	33	33	28.9 (3.9)		15/18	16/17	NR	NR	NR	NR	25.48 (5.35) min	22.43 (5.31)
Khan et al. 2002 [21]	37	30	22.5 (4.7)	22.1 (4.3)	11/26	12/18	NR	NR	NR	NR	NR	NR
Malmstrom et al., 1999 [22]	91	46	23 (4)	22.6 (4.2)	34/57	14/32	NR	NR	49/42	25/21	16 (7)	18 (8)
Malmstrom et al., 2002 [23]	241	45	22.1 (5.1)	23 (6.8)	173/68	30/15	NR	NR	188/53	37/8	NR	NR
Zamiri et al., 2009 [24]	15	14	25 (NR)	26 (NR)	10/5	9/5	NR	NR	NR	NR	10.67 (NR)	8 (NR)

All included studies were assessed using the RoB 2 tool. All 10 RCTs were judged to have a low risk of bias across all five domains (Figure 2).

**Figure 2:**
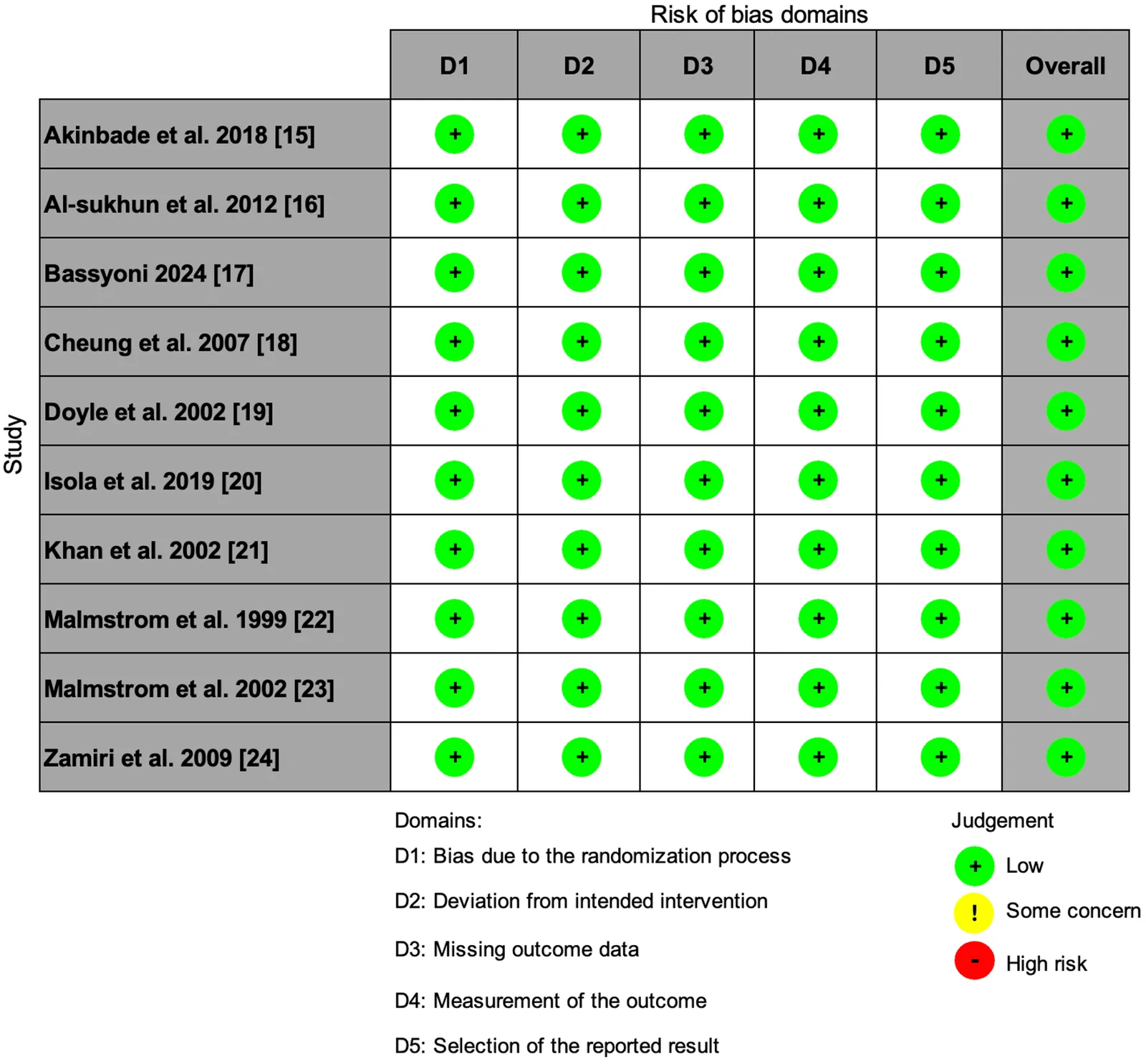
Risk-of-bias assessment

Primary Outcomes

Pain scores at 4-8 hours following third-molar surgery were evaluated using three different pain assessment scales: VAS, TOPAR/TOTPAR, and SPRID/SPID. No statistically significant difference in postoperative pain was observed between the celecoxib and ibuprofen groups on the VAS (SMD = -0.13, 95% CI -0.40 to 0.13; p = 0.33), TOPAR/TOTPAR (SMD = -0.01, 95% CI -0.51 to 0.49; p = 0.97), or SPRID/SPID (SMD = -0.95, 95% CI -2.44 to 0.55; p = 0.21) (Figure 3).

**Figure 3:**
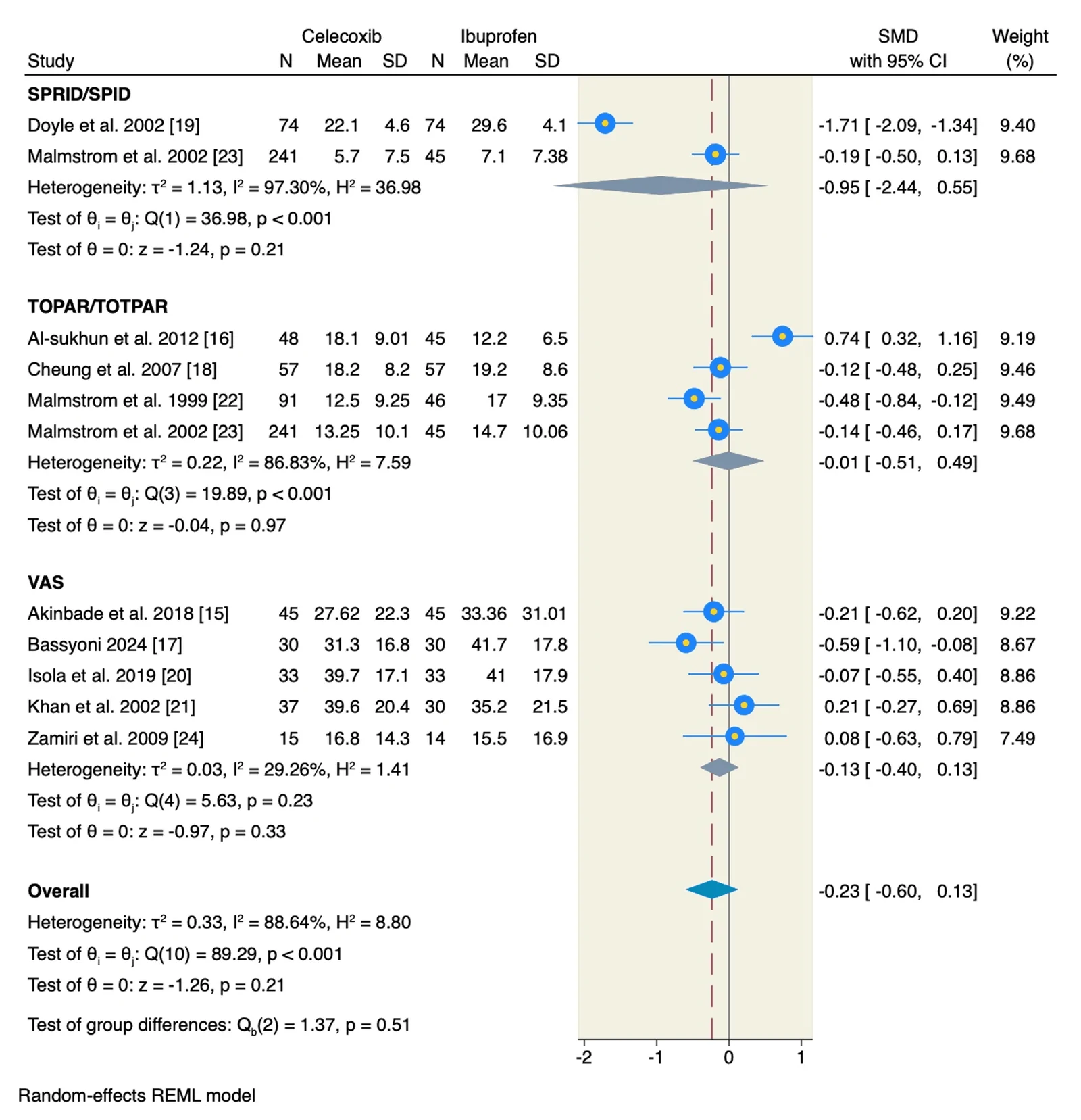
Random-effects model of pain scores at 4-8 hours.

TSA was performed to assess the conclusiveness of the cumulative evidence for pain scores at 4-8 hours (Figure 4). The cumulative Z-curve crossed the conventional statistical significance boundary but did not cross the sequential monitoring boundary for benefit. The cumulative evidence therefore did not meet the predefined TSA criteria for establishing a conclusive beneficial effect of either treatment.

**Figure 4:**
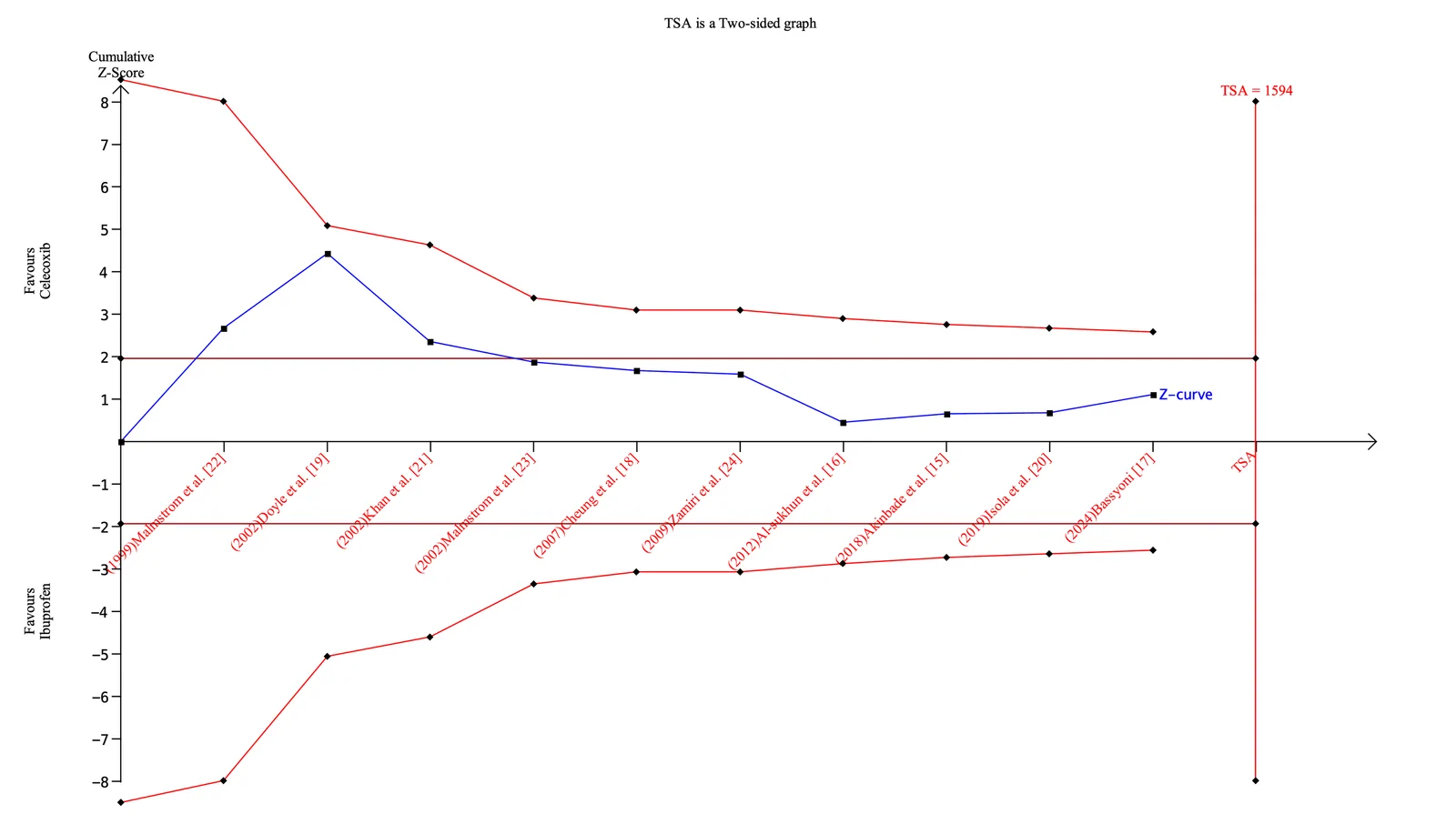
TSA plot of pain scores at 4-8 hours. The cumulative Z-curve crossed the conventional statistical significance boundary but did not cross the sequential monitoring boundary for benefit. The cumulative evidence therefore did not meet the predefined TSA criteria for establishing a conclusive beneficial effect of either treatment. TSA, trial sequential analysis

Pain scores at 12-16 hours were similarly evaluated using the VAS, TOPAR/TOTPAR, and SPRID/SPID scales. No statistically significant difference was observed between celecoxib and ibuprofen when pain was assessed using the VAS (SMD = -0.12, 95% CI -0.39 to 0.14; p = 0.37). Similarly, no significant difference was observed using TOPAR/TOTPAR (SMD = 0.20, 95% CI -0.38 to 0.78; p = 0.50) or SPRID/SPID (SMD = -0.96, 95% CI -2.71 to 0.79; p = 0.28) (Figure 5).

**Figure 5:**
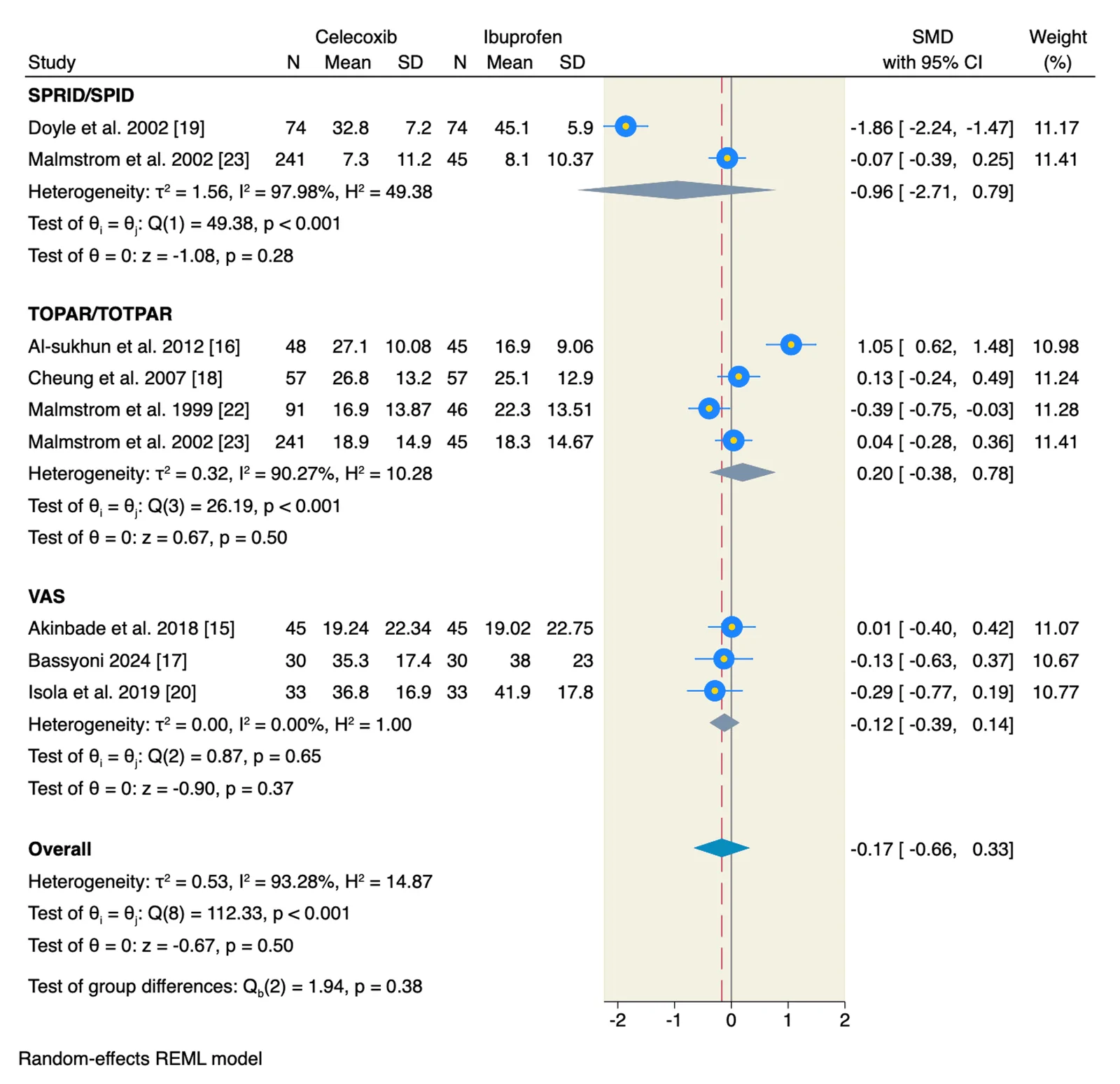
Random-effects model of pain scores at 12-16 hours.

At 24 hours following surgery, pain outcomes were assessed using the VAS and TOPAR/TOTPAR scales. Celecoxib was associated with significantly lower pain scores compared with ibuprofen when assessed using the VAS (SMD = -0.35, 95% CI -0.61 to -0.08; p = 0.01), favoring celecoxib. In contrast, no statistically significant difference was observed between the two treatment groups when pain relief was assessed using TOPAR/TOTPAR (SMD = 0.36, 95% CI -0.00 to 0.73; p = 0.05) (Figure 6).

**Figure 6:**
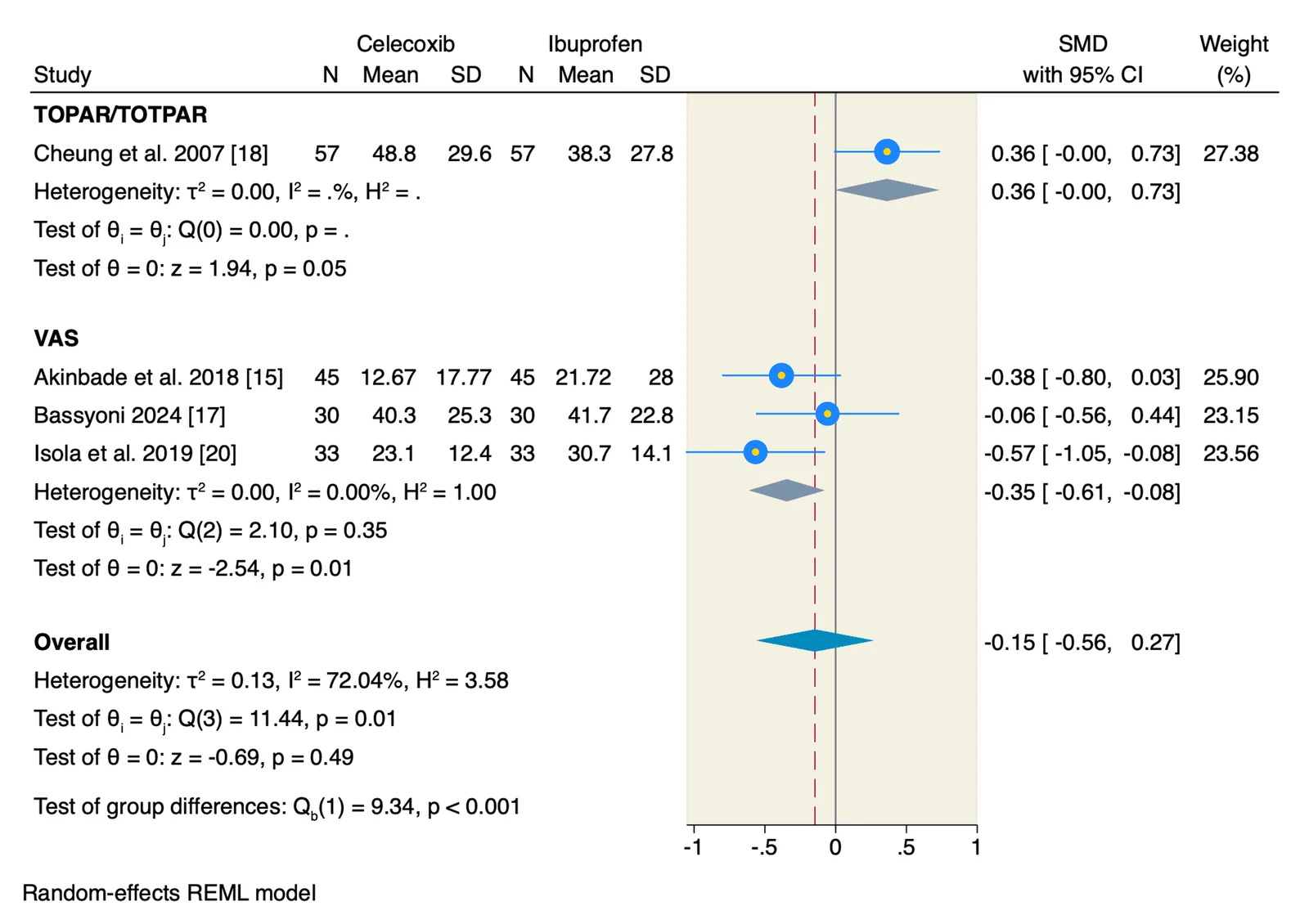
Random-effects model of pain scores at 24 hours.

Secondary Outcomes

The requirement for rescue analgesic medication was reported across the included studies. No statistically significant difference was observed in the number of patients requiring rescue medication between the celecoxib and ibuprofen groups (RR = 1.00, 95% CI 0.8 to 1.25; p = 1.00). These findings indicate that the need for additional analgesic medication was comparable between the two treatment groups (Figure 7).

**Figure 7:**
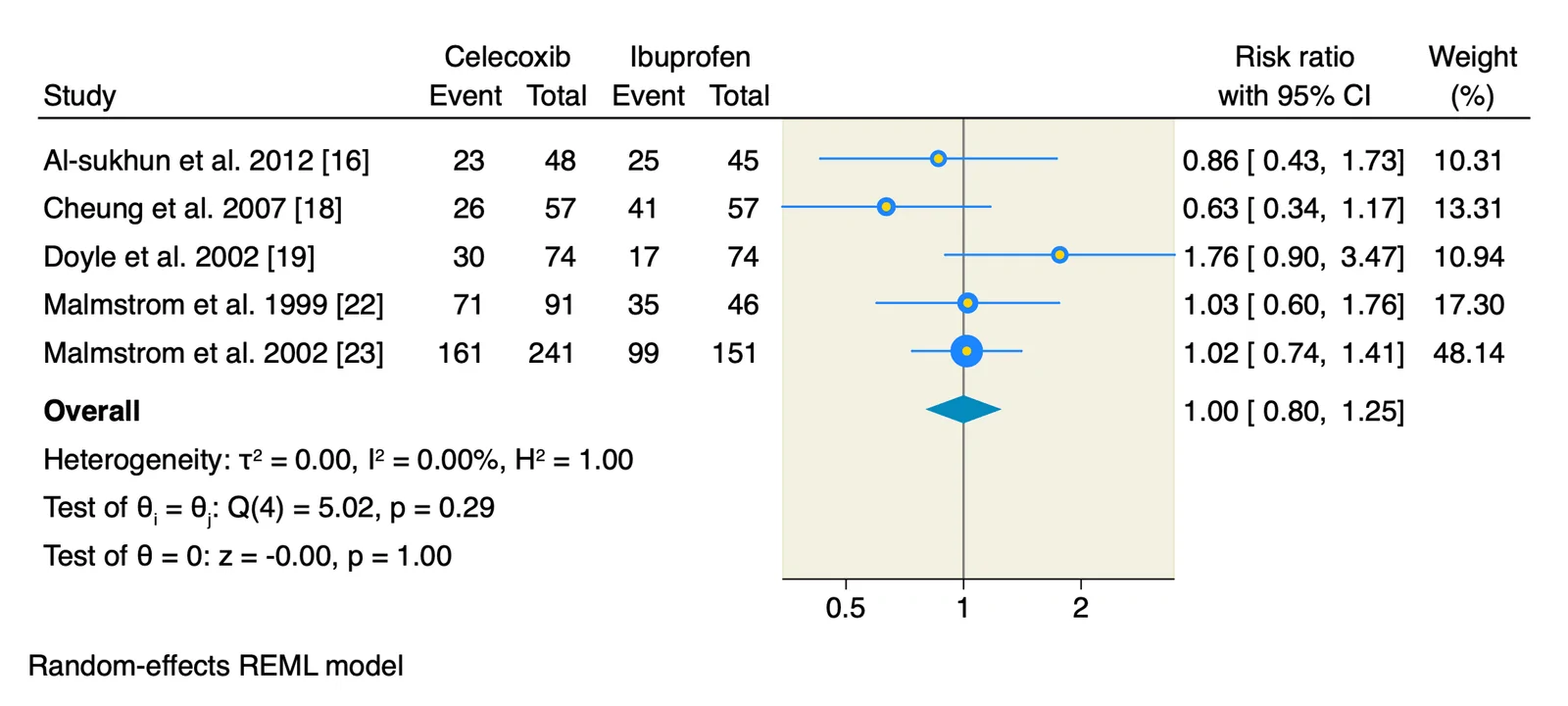
Random-effects model of the number of patients who used rescue medication.

The occurrence of adverse events was also compared between celecoxib and ibuprofen. No statistically significant difference was observed in the number of patients experiencing adverse events between the two groups (RR = 0.93, 95% CI 0.65 to 1.32; p = 0.67). Overall, the available evidence did not demonstrate a significant difference in the incidence of adverse events between celecoxib and ibuprofen (Figure 8).

**Figure 8:**
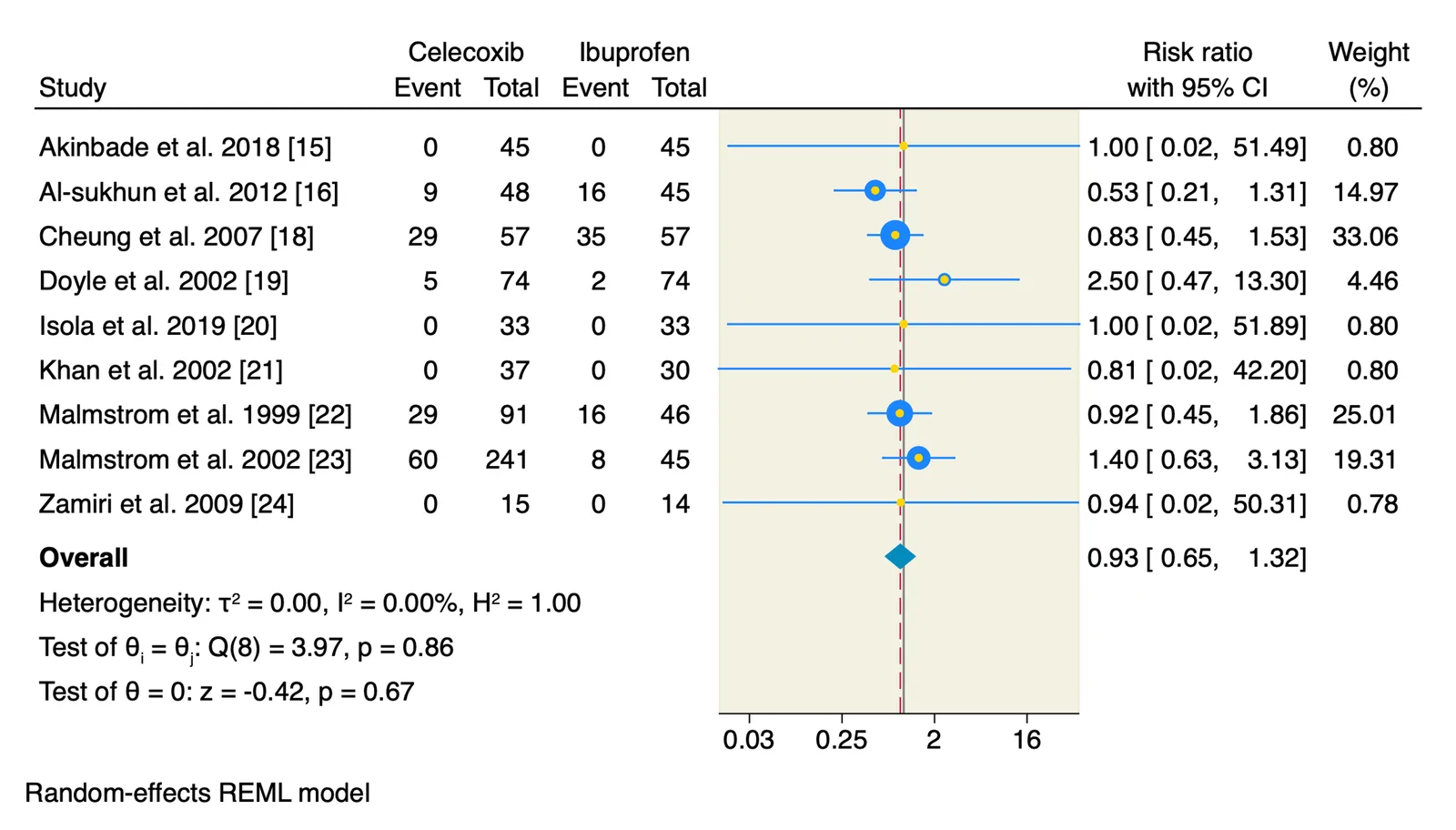
Random-effects model of the number of patients who experienced an adverse event.

Discussion

Main Findings

This systematic review and meta-analysis evaluated the comparative analgesic efficacy and tolerability of celecoxib and ibuprofen following surgical extraction of impacted mandibular third molars. Across 10 RCTs involving 1,090 patients, no significant difference in postoperative pain was observed between the two treatments at 4-8 or 12-16 hours, regardless of whether pain was assessed using VAS, TOPAR/TOTPAR, or SPRID/SPID. At 24 hours, celecoxib was associated with lower pain scores on the VAS, although this difference was not reproduced when analgesic efficacy was assessed using TOPAR/TOTPAR. Similarly, no significant differences were observed in the requirement for rescue analgesic medication or the overall incidence of adverse events. Taken together, these findings suggest that celecoxib and ibuprofen provide broadly comparable postoperative analgesia, with a possible advantage for celecoxib in pain intensity at 24 hours that should be interpreted cautiously.

Comparison With Prior Evidence

The present findings are broadly consistent with the established role of NSAIDs in the management of acute postoperative dental pain. Previous evidence has demonstrated that NSAIDs provide effective analgesia after third-molar surgery, supporting their use as a cornerstone of postoperative pain management [5,25]. However, evidence specifically comparing selective COX-2 inhibition with conventional nonselective NSAID therapy has been less consistent [26]. A previous meta-analysis evaluating celecoxib against non-opioid active controls reported significant benefits in pain intensity, total pain relief, and rescue analgesic use, and suggested a lower frequency of some gastrointestinal adverse effects with celecoxib [27]. While these findings support the potential analgesic advantages of celecoxib, that review included comparisons with several active analgesics rather than being restricted to direct celecoxib-versus-ibuprofen comparisons. The present analysis therefore provides a more focused assessment of the clinical question of whether celecoxib offers superior analgesia to ibuprofen specifically after third-molar surgery. Our findings suggest that such superiority cannot be consistently demonstrated across postoperative time points and pain assessment methods.

Interpretation of Postoperative Pain Outcomes

The lack of a significant difference during the early postoperative period is clinically relevant. The first several hours after third-molar extraction represent a period of substantial inflammatory activity and are often associated with the greatest acute pain burden [28]. Despite the pharmacological differences between selective COX-2 inhibition with celecoxib and nonselective cyclooxygenase inhibition with ibuprofen, neither treatment demonstrated a consistent advantage during this period. This may indicate that both agents provide effective suppression of the inflammatory pathways contributing to early postoperative pain [29]. Differences in dosing regimen, timing of administration, surgical trauma, and baseline pain intensity may also influence the magnitude and timing of analgesic effects across individual trials [17,25].

An important finding of the present analysis was the difference observed at 24 hours when pain was measured using the VAS. Celecoxib was associated with a statistically significant reduction in pain compared with ibuprofen, with an SMD of −0.35. However, this finding was not accompanied by a statistically significant difference in TOPAR/TOTPAR. The discordance between these measures warrants cautious interpretation. VAS primarily reflects pain intensity at specific assessment points, whereas TOPAR/TOTPAR incorporates pain relief over a period of time and may therefore capture a broader aspect of the analgesic experience [30]. The isolated significance observed with VAS at 24 hours may therefore represent a potential late postoperative advantage of celecoxib rather than evidence of consistent superiority across the overall postoperative course. Importantly, the absence of a corresponding reduction in rescue analgesic requirements further argues against interpreting the 24-hour VAS finding as a definitive clinically meaningful advantage.

Clinical Relevance of Pain and Rescue Analgesic Findings

The use of different pain assessment scales is an additional consideration when interpreting the available evidence. Pain intensity, pain relief, and composite measures such as TOPAR/TOTPAR and SPRID/SPID capture related but distinct dimensions of analgesic response [13,31]. In the present analysis, treatment effects were generally consistent in showing no significant difference between celecoxib and ibuprofen at 4-8 and 12-16 hours across the three assessment approaches. This consistency strengthens the conclusion that neither treatment demonstrated a clear early postoperative advantage. Conversely, the differing results at 24 hours highlight the importance of considering the measurement instrument when interpreting pooled analgesic outcomes. Meta-analyses relying exclusively on a single pain scale may therefore provide an incomplete representation of treatment effects.

The lack of a significant difference in rescue analgesic use provides an important clinical context for the pain findings. Although celecoxib demonstrated lower VAS scores at 24 hours, patients receiving celecoxib were not significantly less likely to require additional analgesic medication. Rescue medication use represents a pragmatic measure of whether differences in reported pain translate into a meaningful need for additional treatment [32,33]. The absence of such a difference suggests that the observed reduction in VAS scores at 24 hours may not translate into a substantial difference in overall analgesic requirements. From a clinical perspective, this supports the interpretation that celecoxib and ibuprofen remain broadly comparable options for postoperative pain control following third-molar surgery [20].

Safety and Trial Sequential Analysis

Safety outcomes also did not differ significantly between the two treatments. The pooled analysis showed no significant difference in the number of patients experiencing adverse events. This finding is relevant because the selective COX-2 mechanism of celecoxib is often proposed to provide an improved gastrointestinal tolerability profile by reducing COX-1 inhibition [34]. Although previous evidence has suggested fewer gastrointestinal symptoms with celecoxib in some postoperative dental populations [35], the present analysis did not demonstrate a significant difference in overall adverse events. This may partly reflect the relatively short duration of treatment and the generally healthy, young populations undergoing third-molar surgery. In addition, the included trials were unlikely to be sufficiently powered to detect uncommon clinically important NSAID-related complications.

The findings from the TSA further support a cautious interpretation of the available evidence. Although conventional meta-analysis provides an estimate of the observed treatment effect, repeated testing as additional trials accumulate can increase the probability of random error [36]. In the present analysis, the cumulative evidence did not cross the predefined sequential monitoring boundary for benefit at the assessed time point. Thus, the available sample size and accumulated information were insufficient to establish a conclusive beneficial effect of either treatment. These findings are particularly important when interpreting isolated statistically significant results, as statistical significance in a conventional meta-analysis does not necessarily indicate that the accumulated evidence has reached a level sufficient to exclude random error.

Clinical Implications

From a clinical perspective, the findings suggest that celecoxib should be considered a reasonable alternative to ibuprofen rather than a consistently superior replacement. Ibuprofen remains an effective and widely established analgesic following third-molar surgery, while celecoxib may offer a potential advantage in pain intensity during the later postoperative period [15]. The choice between the two agents may therefore reasonably consider individual patient characteristics, contraindications, dosing preferences, and expected tolerability rather than relying solely on a presumed difference in analgesic efficacy. Importantly, the present findings do not support routine preferential use of celecoxib over ibuprofen solely on the basis of superior postoperative pain control.

Limitations

Several limitations should be considered. First, only 10 RCTs met the eligibility criteria, limiting the statistical power of some pooled analyses and subgroup comparisons. Second, the included studies differed in celecoxib and ibuprofen dosing regimens, timing of administration, and duration of treatment, which may contribute to clinical heterogeneity. Third, surgical characteristics and postoperative pain assessment schedules were not completely uniform across studies. Fourth, different instruments were used to assess analgesic efficacy, and some outcomes were available from a relatively limited number of studies. Fifth, the included populations were generally young and healthy, which may limit the generalizability of the findings to older patients or individuals with substantial comorbidity. Another limitation is the variability in celecoxib and nonselective NSAID dosing regimens across the included trials, ranging from single perioperative doses to repeated administration over several days. Consequently, pain outcomes assessed at the same nominal time point (e.g., 24 hours) may reflect different pharmacological exposure scenarios, particularly when comparing single-dose regimens with multi-day treatment protocols. Although this clinical heterogeneity could not be explored through a sensitivity analysis restricted to single-dose regimens because of the limited number of eligible studies, this variation should be considered when interpreting the pooled estimates. The evidence for the statistically significant reduction in VAS at 24 hours was based on only three RCTs. The limited number of contributing studies and available information also precluded a reliable TSA for this outcome; therefore, the possibility of a chance finding cannot be completely excluded, and this result should be interpreted cautiously. Finally, the relatively small number of studies and short follow-up periods limit the ability to draw firm conclusions regarding uncommon adverse events or longer-term tolerability.

Future Research

Future randomized trials should prioritize direct, adequately powered comparisons of standardized celecoxib and ibuprofen regimens following third-molar surgery. Standardization of dosing, timing of administration, surgical characteristics, rescue analgesic protocols, and pain assessment would facilitate more reliable comparisons across studies. Future research should also incorporate validated and complementary measures of pain intensity and analgesic relief and should be sufficiently powered to evaluate clinically relevant adverse events. Such studies may help determine whether the apparent late postoperative advantage of celecoxib observed in the present analysis represents a reproducible treatment effect or reflects differences in outcome measurement and study characteristics.

## Conclusions

This systematic review and meta-analysis of 10 RCTs including 1,090 patients found that celecoxib and ibuprofen provide broadly comparable analgesic efficacy following third-molar surgery. Although celecoxib was associated with lower VAS pain scores at 24 hours, no consistent differences were observed across other pain assessment measures, rescue analgesic use, or adverse events. Overall, the available evidence does not establish celecoxib as superior to ibuprofen, supporting both agents as effective options for postoperative pain management.

## Appendices

**Table 3:** Detailed search strategy of each database.

Database	Search strategy	Filters	Results
PubMed	((celecoxib OR ibuprofen OR NSAIDs OR cyclooxygenase inhibitors OR COX-2 inhibitors OR non-steroidal anti-inflammatory drugs OR anti-inflammatory agents, non-steroidal) AND (third molar surgery OR third molar extraction OR wisdom tooth extraction OR impacted third molar OR mandibular third molar OR molar, third) AND (postoperative pain OR pain management OR analgesia OR pain relief OR VAS OR visual analogue scale OR pain, postoperative))	English Language	N = 561
Scopus	(TITLE-ABS-KEY (“celecoxib” OR “ibuprofen” OR “nsaids” OR “cyclooxygenase inhibitor” OR “cox-2 inhibitor” OR “non-steroidal anti-inflammatory drug”) AND TITLE-ABS-KEY (“third molar surgery” OR “third molar extraction” OR “wisdom tooth extraction” OR “impacted third molar” OR “mandibular third molar”) AND TITLE-ABS-KEY (“postoperative pain” OR “pain management” OR “analgesia” OR “pain relief” OR “vas” OR “visual analogue scale”))	English language	N = 409
Web of Science	(((TS=(celecoxib OR ibuprofen OR NSAIDs OR cyclooxygenase inhibitors OR COX-2 inhibitors OR non-steroidal anti-inflammatory drugs)) AND TS=(third molar surgery OR third molar extraction OR wisdom tooth extraction OR impacted third molar OR mandibular third molar)) AND TS=(postoperative pain OR pain management OR analgesia OR pain relief OR VAS OR visual analogue scale))	English language	N = 383
Cochrane CENTRAL	(celecoxib OR ibuprofen OR NSAIDs OR cyclooxygenase inhibitors OR COX-2 inhibitors OR non-steroidal anti-inflammatory drugs) AND (third molar surgery OR third molar extraction OR wisdom tooth extraction OR impacted third molar OR mandibular third molar) AND (postoperative pain OR pain management OR analgesia OR pain relief OR VAS OR visual analogue scale)	English language	N = 636
